# LH-21, A Peripheral Cannabinoid Receptor 1 Antagonist, Exerts Favorable Metabolic Modulation Including Antihypertensive Effect in KKAy Mice by Regulating Inflammatory Cytokines and Adipokines on Adipose Tissue

**DOI:** 10.3389/fendo.2018.00167

**Published:** 2018-04-20

**Authors:** Ziqi Dong, Hui Gong, Yadan Chen, Hong Wu, Jun Wu, Yinghong Deng, Xinmao Song

**Affiliations:** ^1^Department of Cardiology, Jinshan Hospital of FuDan University, Shanghai, China; ^2^Department of Pharmacy, The Second Hospital of Jilin University, Changchun, China; ^3^Department of Radiation Oncology, Eye, Ear, Nose & Throat, Hospital of FuDan University, Shanghai, China

**Keywords:** cannabinoid receptor 1, obesity, hypertension, adipokine, inflammation

## Abstract

Patients with obesity are susceptible to hypertension and diabetes. Over-activation of cannabinoid receptor 1 (CB_1_R) in adipose tissue is proposed in the pathophysiology of metabolic disorders, which led to the metabolic dysfunction of adipose tissue and deregulated production and secretion of adipokines. In the current study, we determined the impact of LH-21, a representative peripheral CB_1_R antagonist, on the obesity-accompanied hypertension and explored the modulatory action of LH-21 on the adipose tissue in genetically obese and diabetic KKAy mice. 3-week LH-21 treatment significantly decreased blood pressure with a concomitant reduction in body weight, white adipose tissue (WAT) mass, and a slight loss on food intake in KKAy mice. Meanwhile, glucose handling and dyslipidemia were also markedly ameliorated after treatment. Gene expression of pro-inflammatory cytokines in WAT and the aortae were both attenuated apparently by LH-21, as well the mRNA expression of adipokines (lipocalin-2, leptin) in WAT. Concomitant amelioration on the accumulation of lipocalin-2 was observed in both WAT and aortae. In corresponding with this, serum inflammatory related cytokines (tumor necrosis factor α, IL-6, and CXCL1), and lipocalin-2 and leptin were lowered notably. Thus according to current results, it can be concluded that the peripheral CB_1_R antagonist LH-21 is effective in managing the obesity-accompanied hypertension in KKAy mice. These metabolic benefits are closely associated with the regulation on the production and secretion of inflammatory cytokines and adipokines in the WAT, particularly alleviated circulating lipocalin-2 and its accumulation in aortae.

## Introduction

Obesity, especially abdominal obesity, is closely associated with a variety of metabolic disorders particularly hypertension, type 2 diabetes, and dyslipidemia. Thus, an effective anti-obesity molecule with a concomitant improvement in metabolic profile is essential in hypertension with visceral obesity. Over-activation of endocannabinoids and increased expression of cannabinoid receptor 1 (CB_1_R) in adipose tissue had been confirmed under condition of morbid obesity (1, 2), and CB_1_R had been proved to be an attractive target in developing novel anti-obesity drugs (3–6). Among them, the second generation of peripheral CB_1_R-targeted neutral antagonist or inverse agonist with limited access to the central nervous system is holding great promise to be the next blockbuster for obesity therapy. They are assumed to avoid the psychiatric adverse effects that had led to the withdrawn of rimonabant, the first in-class CB_1_R inverse agonist and antagonist, from the market (7, 8). Recent studies indicated that the beneficial actions of CB_1_R blockage in decreasing body fat and improving insulin resistant were in close association with the improvement on the concentrations of local and systemic adipokines. Such as rimonabant (SR141716) was demonstrated to be able to increase level of serum adiponectin, decrease serum tumor necrosis factor α (TNFα) and leptin content, as well as upregulate the expression of adiponectin in adipose tissue (9, 10). LH-21, 5-(4-chlorophenyl)-1-(2,4-dichlorophenyl)-3-hexyl-1H-1,2,4-triazole, was a recently discovered representative peripheral CB_1_R-targeted neutral antagonist or weak inverse agonist (11), and it had displayed sustained body weight reduction and improvement in insulin sensitivity in genetic obese Zucker rat and dietary induced obese rodent models (12, 13). However, whether peripheral CB_1_R-targeted antagonist is effective in managing the obesity-accompanied hypertension is unknown.

Accumulating evidence indicates adipose tissue regulates systemic energy homeostasis and insulin sensitivity through controlling the production and secretion of a series of adipokines (14–16). Among these adipose tissue-derived cytokines, adiponectin was proved to be able to promote vasodilatation and improve endothelial function, whereas some adipokines, such as pro-inflammatory adipokine lipocalin-2, could aggravate obesity-induced endothelial dysfunction and vascular inflammation, and had pleiotropic pathogenic roles in obesity-associated metabolic disorders and hypertension (17, 18). Lipocalin-2 is produced by white adipose tissue (WAT). Serum concentration of lipocalin-2 is significantly elevated in obese humans and animals, and positive correlation of it with body fat, arterial blood pressure, insulin resistance index, and abnormal lipid profiles had been established (19–21). Administration of lipocalin-2 could promote endothelial dysfunction, induce adipose tissue inflammation, and cause abnormal vasodilator response in high-fat diet-induced obese mice (22). By contrast, deficiency of lipocalin-2 protects against dietary obesity-induced endothelial and cardiometabolic dysfunctions (22–24). Thus, a molecule that could regulate the production and secretion of lipocalin-2 might ameliorate the deteriorated endothelial and metabolic dysfunctions induced by obesity, and finally contribute to the amelioration of obesity-accompanied hypertension.

To explore if the peripheral CB_1_R antagonist LH-21 will be of benefit to the management of obesity-accompanied hypertension, and further to clarify the underlying mechanism, the impact of LH-21 on obesity-accompanied hypertension was investigated in KKAy mice, a polygenic mouse model of human obesity, T2DM, dyslipidemia, and hypertension (25–27). The KK-Ay mice are spontaneous heterozygous diabetic mice and a congenic strain in which the yellow obese Ay allele at the mouse agouti locus of the C57BL/6J-Ay strain was transferred to the inbred KK strain by repetitive backcrossing (28, 29). Body weight gain, food intake, glucose homeostasis, and blood pressure were examined after 3-week LH-21 treatment. Gene expression of pro-inflammatory cytokines in adipose tissue and aortae, and gene expression of adipokines in adipose tissue was measured, and serum concentration of adipokines was assayed.

## Materials and Methods

### Animal Studies

8-week-old female C57BL/6J mice (NC mice) and spontaneous diabetic KKAy mice were purchased from the Experimental Animal Center, Chinese Academy of Medical Sciences, Beijing. Mice were housed in a room under controlled temperature (23 ± 1°C) and 12-h light–dark cycle. The KKAy mice were fed with a high-fat diet and water *ad libitum*. At the beginning of the study, the KKAy mice were randomized into the vehicle-treated model control group (MC) or the two LH-21 (1 and 3 mg/kg) treatment groups based on their initial body weight and blood glucose levels (*n* = 8). LH-21 (Cayman Chemical, Ann Harbor, MI, USA) was administered by IP injection daily for 3 weeks, while mice in NC and MC group were IP injected with vehicle (5% Tween 80 and 1% ethanol) for 3 weeks. The dosages of LH-21 were chosen according to previously described (12, 30). Individual body weight and food consumption were measured every 2 days. At the last day of the experiment, overnight fasted mice were sacrificed by decapitation. Plasma was collected for immediate assessment of serum biochemical parameters. The intraperitoneal WAT and interscapular brown adipose tissue (BAT) were excised and weighed, and then the WAT and the isolated aortae were rapidly frozen in liquid nitrogen for subsequent gene expression and western blot assay.

### Glucose Tolerance and Insulin Sensitivity Assessment

At 16th day of treatment, oral glucose tolerance test (OGTT) was performed on KKAy and C57BL/6J control mice after overnight fast. A solution of 20% glucose (Sigma Aldrich, St Louis, MO, USA) was gavage orally at a dose of 1.5 g/kg. Tail bleeds were taken for whole blood glucose readings using One-Touch glucometers (Johnson & Johnson, USA) at 0, 30, 60, and 120 min. The area under the curve (AUC) of OGTT generated from blood glucose recordings was calculated. The insulin sensitivity index (ISI) was calculated from the values of fasting blood glucose (FBG) and fasting blood insulin (FBI). ISI was calculated as: 1/(FBG × FBI) × 1,000 (29, 31).

### Biochemistry Analysis

Serum level of total cholesterol (TC), triglycerides (TG), and free fatty acid (FFA) were determined by using enzymatic colorimetric method with commercial kits according to the manufacturer’s instructions (Rongsheng Biotech, Shanghai China). Serum lipocalin-2 level was measured using ELISA method (17). Serum level of insulin, leptin, IL-6, TNFα, and C-X-C motif ligand 1 (CXCL1) concentration were assayed by using MILL IPLEX MAP Mouse Metabolic Magnetic Bead Panel kits (Millipore, MA, USA) with FlexMAP3D. Serum high molecular weight adiponectin was determined with commercial ELISA kit (ALPCO Diagnostics, USA).

### QPCR Analysis

Total RNA from the intraperitoneal adipose tissue or the aortae of the mice were prepared with the Trizol RNA preparation kit following the manufacturer’s recommended procedures (Gibco-BRL, Grand Island, NY, USA). The quality and integrity of RNA were guaranteed by the ratio of 260/280 (between 1.8 and 2.0) and agarose gel electrophoresis before converted to cDNA with oligo dT primers by using a cDNA synthesis kit (Takara Biotechnology Co. Ltd., Dalian, China) in a thermocycler (Mastercycler, Eppendorf, Hamburg, Germany). Quantitation of target genes was performed using SYBR Green PCR Master Mix (Applied Biosystems, Warrington, UK) with the ABI PRISM 7900 Sequence Detection System (Applied Biosystems, Foster City, CA, USA). The relative amount of all mRNAs was calculated using the comparative CT method (2^−ΔΔCt^). Target gene expression is presented relative to β-actin expression. The primer sequences are listed in Table S1 in Supplementary Material.

### Western Blot Analysis

Tissue lysates from the intraperitoneal adipose tissue or the aortae (30 µg) were resolved by SDS-PAGE, and proteins were then transferred to polyvinylidene difluoride membranes. After blocking with 5% milk blocking buffer (Tris-buffered saline with 0.1% Tween 20), the membranes were incubated overnight at 4°C with antibodies against lipocalin-2 and β-actin (Cell Signaling, Beverly, MA, USA), respectively, and followed by horseradish peroxidase-conjugated secondary antibody. Detection of immunoreactive band was achieved using enhanced chemiluminescence detection reagents (Applygen Technologies Inc., Beijing, China), and scanned on an Alpha Imager 5500 (Alpha Innotech, San Leandro, CA, USA) imaging densitometer. The expression of proteins was normalized to that of β-actin.

### Blood Pressure Measurement

At the 20th day of the experiment, blood pressure and heart rate were measured in conscious mice between 10:00 and 13:00 using the tail-cuff method (MRBP system, IITC Life Science, USA) according to previous description, and at least 8 readings were taken for each detection(27, 32).

### Statistical Analysis

Statistical analyses were assessed by one-way ANOVA followed by the Tukey’s multiple comparison tests with SPSS (SPSS Inc., Chicago, IL, USA) to compare the experimental groups. For all statistical comparisons, a *p* value <0.05 was considered statistically significant.

## Results

### Effects of LH-21 on Metabolic Parameters and Serum Lipids in KKAy Mice

**Figure 1:**
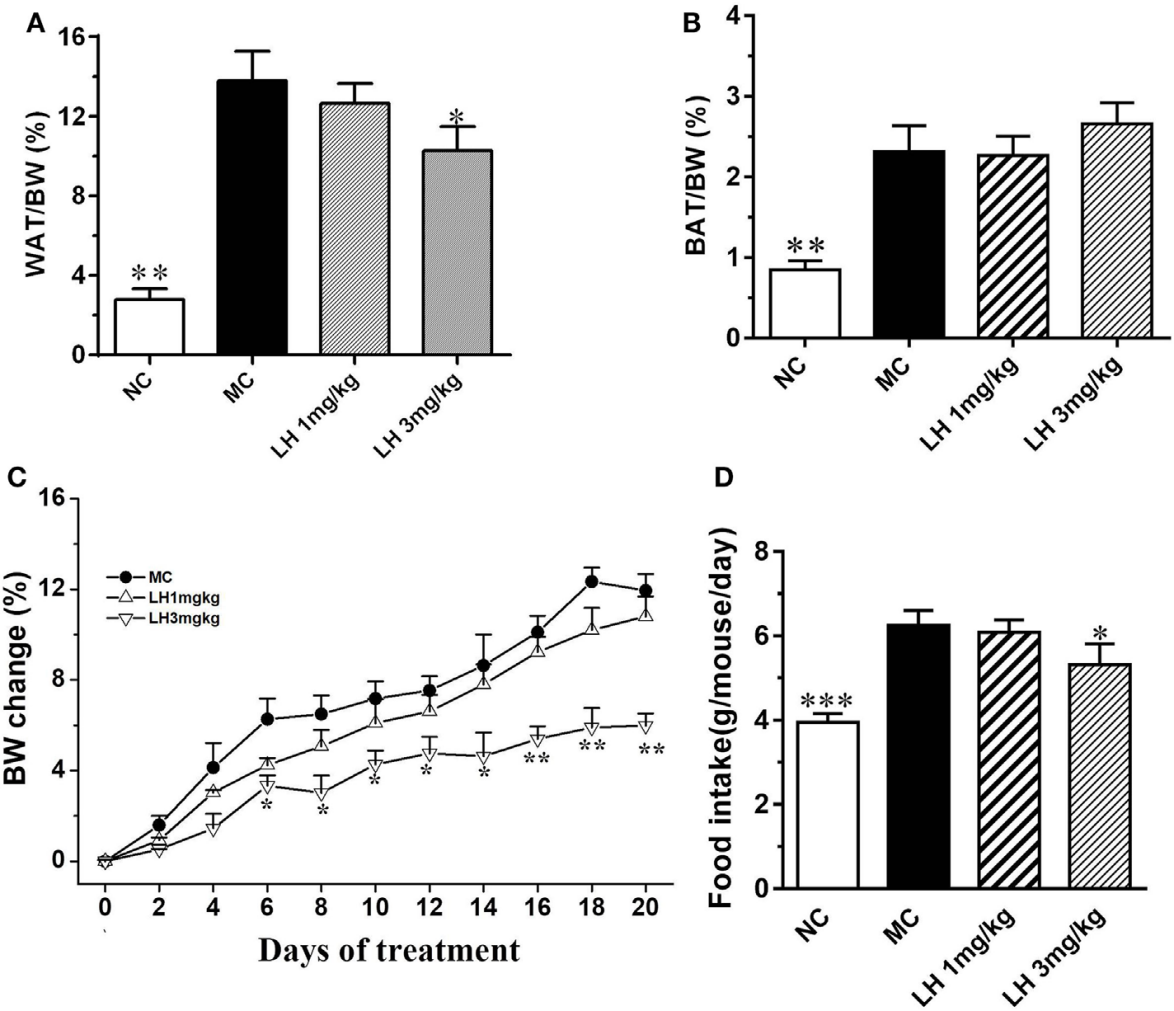
Impact of LH-21 on metabolic parameters in KKAy mice. **(A)** Intraperitoneal adipose tissue weight index. **(B)** BAT weight index. **(C,D)** Body weight change and average food intake during the experiment. The KKAy mice were injected daily with vehicle or LH-21 (LH) for 3 weeks. NC, normal control; MC, model control; WAT, white adipose tissue; BAT, brown adipose tissue; BW, body weight. Values are mean ± SEM. *n* = 8; *p < 0.05, **p < 0.01, ***p < 0.001 vs. MC group.

The body weight of the KKAy mice before treatment was significantly greater than that of the control C57BL/6J mice (41.5 ± 2.8 vs. 25.3 ± 1.4 g, *p* < 0.01). Meanwhile, the KKAy mice are significantly obese, with strikingly accumulated adipose tissues (Figures 1A,B). After treatment, a striking decrease in body weight was observed from the sixth day onward in 3 mg/kg LH-21 group, and lasted till the end of the experiment (Figure 1C). The body weight loss is presumed to be partially associated with the slightly decreased food intake induced by LH-21, as compared to vehicle-treated KKAy mice (3 mg/kg LH-21 vs. MC *p* < 0.01) (Figure 1D). Correspondingly, the WAT mass was reduced significantly (*p* < 0.01), while the BAT mass was not affected (Figures 1A,B). Additionally, 1 mg/kg LH-21 showed no impact on body and fat weight, as well food intake after 3 weeks administration.

**Figure 2:**
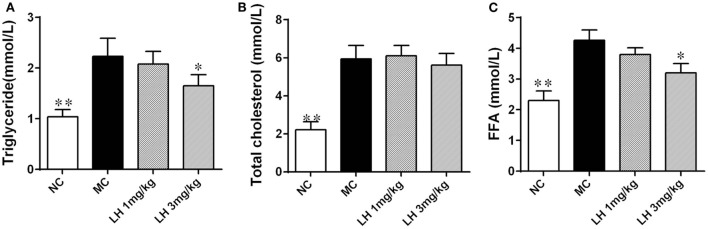
Impact of LH-21 on serum lipids in KKAy mice. **(A)** Triglyceride. **(B)** Total cholesterol. **(C)** Free fatty acid. NC, normal control; MC, model control; LH, LH-21. Values are mean ± SD. *n* = 8; *p < 0.05, **p < 0.01 vs. MC group.

Compared to that of normal C57BL/6J mice, the KKAy mice had significantly elevated serum lipids including TG, TC, and FFA (Figure 2). LH-21 showed dose-dependent effect on serum TG and FFA levels, significant reduction was noticed in the 3 mg/kg group (*p* < 0.05 for both) (Figures 2A,C). However, both dosages of LH-21 showed no impact on serum TC in KKAy mice (Figure 2B).

### Effects of LH-21 on Glucose Metabolism in KKAy Mice

**Figure 3:**
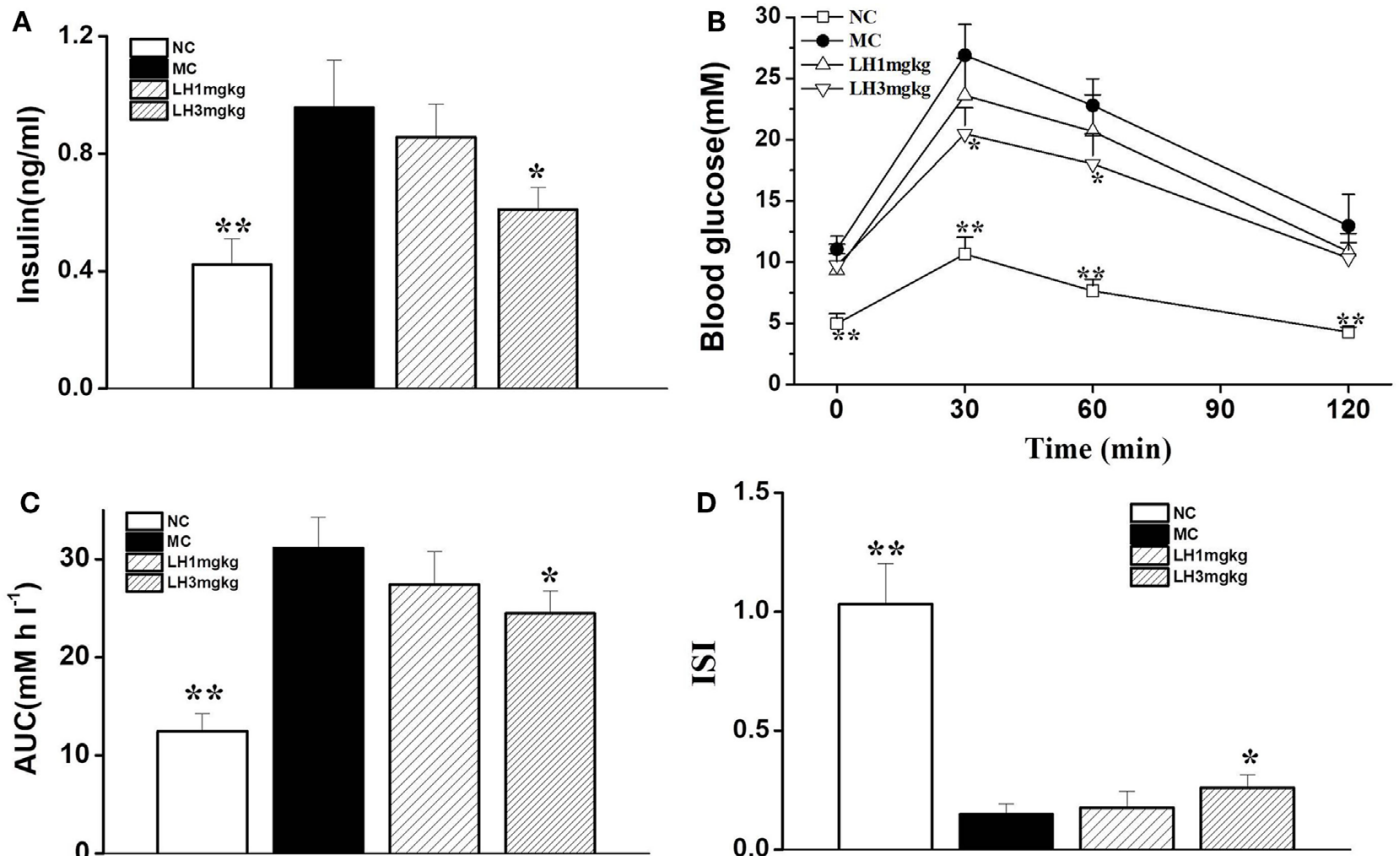
Effect of LH-21 on glucose tolerance and serum insulin level in KKAy mice. **(A)** Fasting serum insulin level. **(B)** Oral glucose tolerance test (OGTT). At the 16 day of treatment, overnight fasted mice were subjected to OGTT, and blood glucose was tested at the indicated time. **(C)** The area under the curve (AUC) of OGTT. **(D)** The insulin sensitivity index (ISI). NC, normal control; MC, model control; LH, LH-21. Values are mean ± SD. *n* = 8; *p < 0.05, **p < 0.01 vs. MC group.

Relative to the normal C57BL/6J mice, the KKAy mice displayed apparent hyperinsulinemia, and increased FBG and impaired glucose tolerance (Figure 3). Daily treatment with LH-21 for up to 3 weeks dose dependently lowered serum insulin and enhanced insulin sensitivity. When compared to the vehicle-injected KKAy mice, the glucose intolerance was markedly restored by 3 mg/kg LH-21 as assessed by the OGTT and the corresponding AUC (Figures 3B,C), significantly decreased glucose values were observed at time point 30 and 60 min after glucose overload (Figure 3B). Moreover, hyperinsulinemia and diminished ISI were also improved strikingly by 3 mg/kg LH-21 (Figures 3A,D).

### Effects of LH-21 on Blood Pressure in KKAy Mice

**Figure 4:**
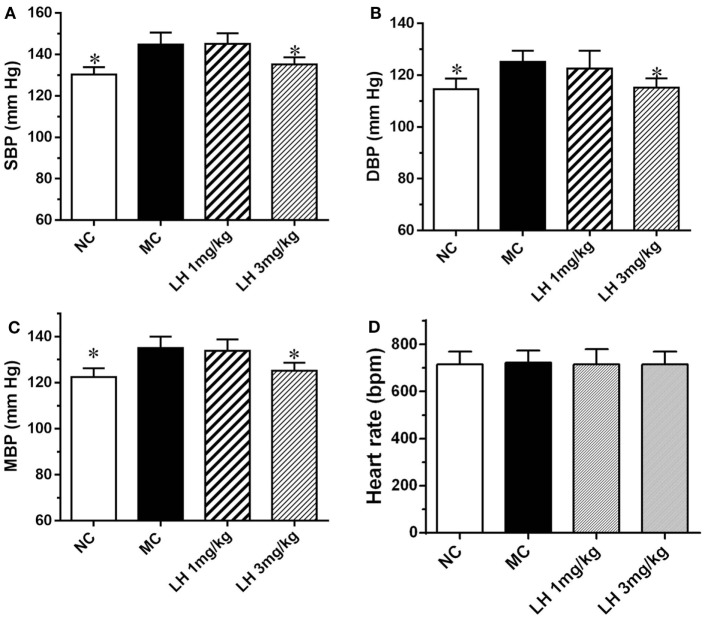
Effect of LH-21 on blood pressure in KKAy mice. **(A)** Systolic blood pressure (SBP). **(B)** Diastolic blood pressure (DBP). **(C)** Mean blood pressure (MBP). **(D)** Heart rate. Blood pressure and heart rate were measured by the tail-cuff method. NC, normal control; MC, model control; LH, LH-21. Values are mean ± SEM. *n* = 8; *p < 0.05 vs. MC group.

We next examined the effects of LH-21 on blood pressure and heart rate in KKAy mice. As shown in Figure 4, compared to the normal C57BL/6J mice, the systolic blood pressure (SBP), diastolic blood pressure (DBP), and mean blood pressure (MBP) of the KKAy mice was elevated by 11.1, 10.3, and 9.2%, respectively (Figure 4). With respect to the effects on blood pressure, significantly lowered SBP, DBP, and MBP were found in 3 mg/kg LH-21-treated group relative to the vehicle-treated KKAy mice (*p* < 0.05), instead of the lower dose (1 mg/kg) LH-21 group (Figures 4A–C). In addition, no difference on heart rate was found among the vehicle and LH-21-treated groups (Figure 4D).

### The Impact of LH-21 on Inflammatory Cytokines and Adipokines in KKAy Mice

**Figure 5:**
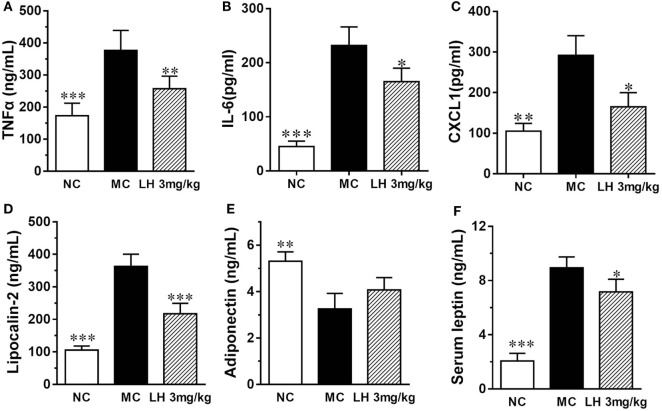
Impact of LH-21 on serum adipokines in KKAy mice. **(A)** TNFα, **(B)** IL-6, **(C)** CXCL1 **(D)** Lipocalin-2, **(E)** Adiponectin, **(F)** leptin. NC, normal control; MC, model control; LH, LH-21. Values are mean ± SD. *n* = 8; *p < 0.05, **p < 0.01, ***p < 0.001 vs. MC group.

Representative inflammatory cytokines, together with the levels of the adipokines lipocalin-2, leptin, and adiponectin were assayed on serum samples from 3 mg/kg LH-21- or vehicle-treated KKAy mice. As shown in Figure 5, when compared with that of NC mice, the serum levels of TNFα, IL-6, and CXCL1 were significantly elevated in the vehicle-treated KKAy mice (Figures 5A–C). Meanwhile, serum adipokines produced by adipose tissues in the KKAy mice were dysregulated, the levels of lipocalin-2 and leptin were strikingly increased relative to that of the NC group (*p* < 0.001) (Figures 5D,F), whereas adiponectin was reduced remarkably (*p* < 0.01) (Figure 5E). However, all the determined inflammatory cytokines in serum were lowered by 3 mg/kg LH-21 markedly (Figures 5A–C). Moreover, both lipocalin-2 and leptin levels were decreased notably after 3 mg/kg LH-21 treatment, whereas no statistical effect on serum adiponectin was detected after treatment (Figures 5D–F).

**Figure 6:**
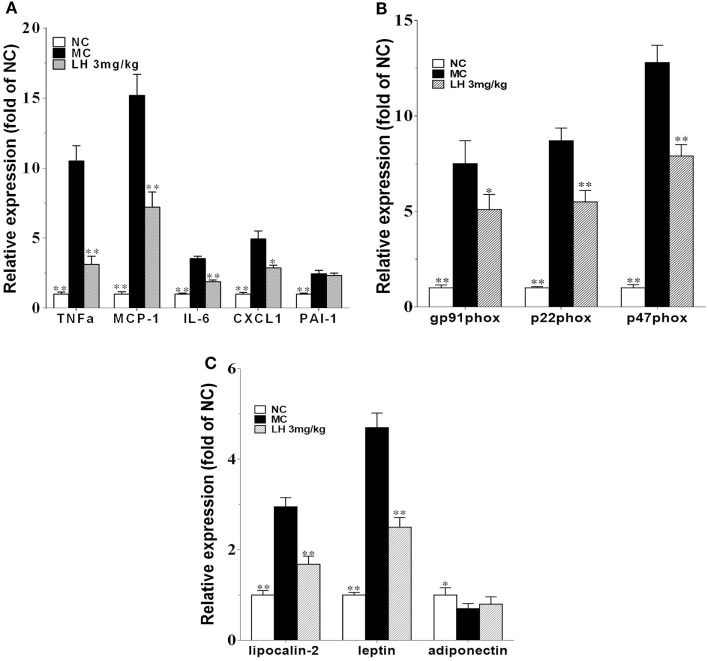
Impact of LH-21 on gene expression in adipose tissue in KKAy mice. mRNA expression of pro-inflammatory cytokines **(A)**, NADPH oxidase components **(B)**, and adipokines **(C)**. Results are normalized to β-actin in the correspondent group, and expressed as relative expression compared with that in the NC group. NC, normal control; MC, model control; LH, LH-21. Values are mean ± SD. *n* = 3; *p < 0.05, **p < 0.01 vs. MC group.

**Figure 7:**
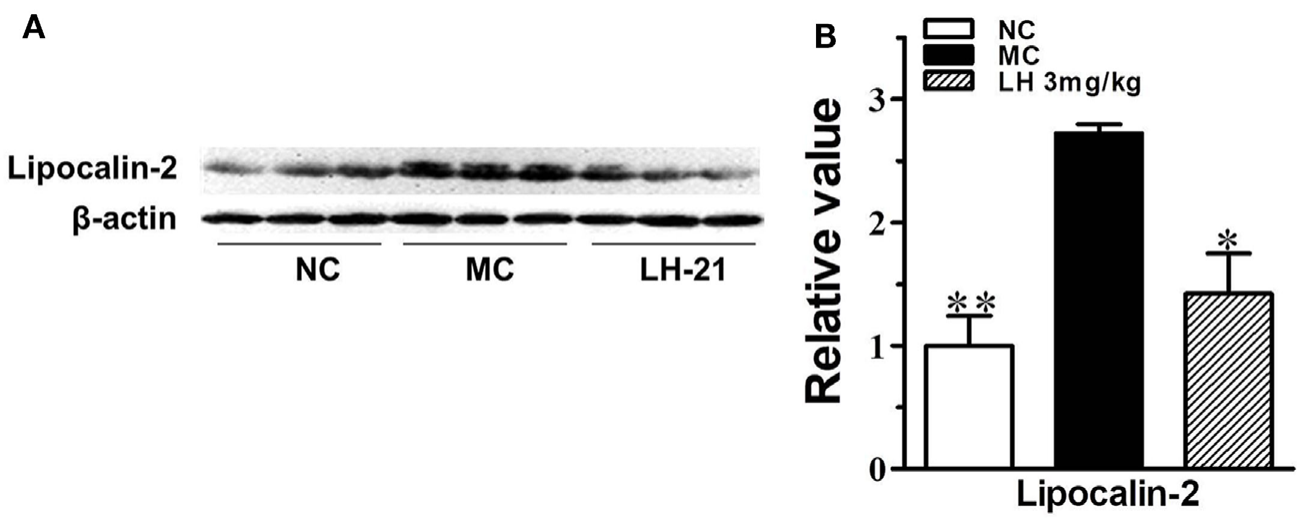
Impact of LH-21 on adipose tissue lipocalin-2 protein expression in KKAy mice. **(A)** Expression of lipocalin-2 in adipose tissue determined by western blot. **(B)** Quantification of the result in panel **(A)**. NC, normal control; MC, model control; LH, LH-21. *n* = 3; values are mean ± SD, *n* = 3; *p < 0.05, **p < 0.01 vs. MC group.

To further investigate the effects of LH-21 on the origin of these inflammatory cytokines and adipokines, we examined their gene expression in intraperitoneal WAT in KKAy mice. The result showed that the expression of monocyte chemoattractant protein 1 (MCP-1), IL-6, TNFα, and CXCL1 mRNA was significantly downregulated in WAT of the LH-21-treated group compared with the vehicle-treated KKAy mice, while the PAI-1 mRNA expression was not altered (Figure 6A). The dysregulated production of adipokines and pro-inflammatory cytokines is reported to activate NADPH oxidase components that function as important intracellular second messengers to modulate endothelial function, and expression of pro-inflammatory mediators. We thus examined gene expression of NADPH oxidase (p22phox, gp91phox, and p47phox) in the WAT. As expected, expression of these NADPH oxidase was markedly elevated (p < 0.01 vs. C57BL/6J) in the WAT of the KKAy mice (Figure 6B). However, they were decreased notably by treatment with 3 mg/kg LH-21. Furthermore, expression of adipokines in WAT was also partially recovered, mRNA levels of lipocalin-2 and leptin were decreased strikingly (*p* < 0.01), whereas adiponectin was slightly increased (Figure 6C). In consistent with the gene expression, protein level of lipocalin-2 in the WAT of 3 mg/kg LH-21-treated KKAy mice was also remarkably reduced after treatment (Figure 7) (*p* < 0.01 vs. MC).

### The Impact of LH-21 on Lipocalin-2 Protein Accumulation in Aortae of KKAy Mice

**Figure 8:**
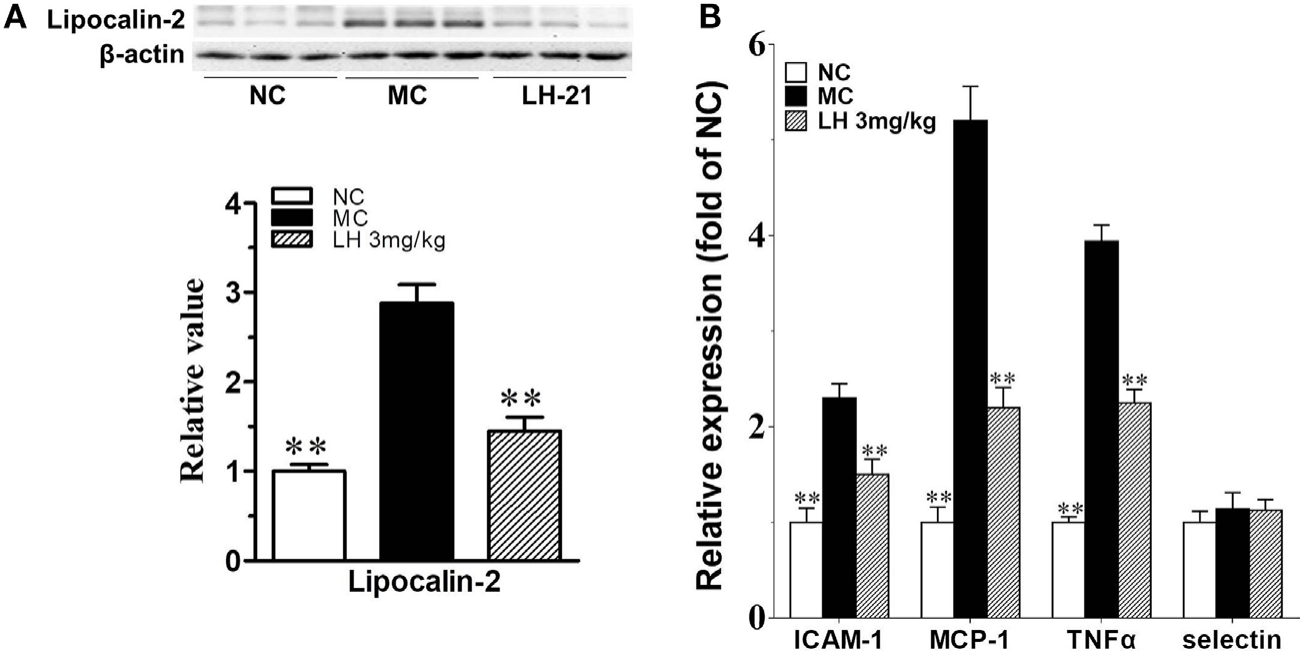
Impact of LH-21 on lipocalin-2 protein expression and gene expression of inflammatory cytokines in the aorta of KKAy mice. **(A)** Lipocalin-2 protein expression determined by western blot and the corresponding quantification result. **(B)** mRNA expression of inflammation related cytokines. Results are normalized to β-actin and expressed as relative expression compared with that in NC group. NC, normal control; MC, model control; LH, LH-21. Values are mean ± SD, *n* = 3; **p < 0.01 vs. MC group.

It had been demonstrated that lipocalin-2 accumulation is significantly increased in aortic tissues of obese mice with elevated blood pressure, which will cause the deterioration of endothelial vasodilator function. Here, we also found lipocalin-2 protein content was significantly increased by about threefold in the aortae of KKAy mice (*p* < 0.01 vs. MC) (Figure 8A). This is in consistent with the elevation of circulating lipocalin-2 content. 3-week treatment with LH-21 effectively decreased the accumulation of lipocalin-2 in aortae (*p* < 0.01 vs. MC) (Figure 8A). In consistent with this, most of the assayed inflammatory cytokines expression in aortae were also downregulated noticeably (Figure 8B).

## Discussion

LH-21 was initially demonstrated to be a silent peripheral CB_1_R antagonist (12, 33). Subsequently, other authors identified LH-21 as a weak inverse CB_1_R agonist at higher dosage (34). However, whether the amounts crossed the blood–brain barrier could induce physiological effects is still in disputation. Herein in the KKAy mice model, we investigated the antihypertension effect of LH-21 and explored the modulatory action of LH-21 to inflammatory cytokines and adipokines. 3-week sub-chronic treatment with 3 mg/kg LH-21 not only significantly reduced the body weight gain and improved glucose handling, but also displayed apparent ability to counteract obesity-related high blood pressure and without affecting heart rate. These metabolic benefits are closely associated with its regulation on the production and secretion of inflammatory cytokines and adipokines from the WAT, besides the slight effect on food consumption (Figure S1 in Supplementary Material).

In agreement with previous studies on the high-fat diet-induced obese rat and the genetic obese Zucker rat (12, 13), here we also found that only the higher dose of LH-21 (3 mg/kg) showed clear anti-obesity action, apparent decrease on body weight occurred after about 1-week administration. On the one hand, this effect is related to the slightly reduced food intake, which may be partially associated with the lowered serum leptin induced by 3 mg/kg LH-21 because elevated plasma leptin is correlated with hyperphagia, insulin resistance, and other constituents of metabolic syndrome (35). However, a CB_1_-independent effect might also play a role in this effect (30, 36). On the other hand, the reduction of LH-21 on weight gain can be attributed to the reduction in energy storage into the adipocytes and an increase in energy expenditure in the WAT, as it had been demonstrated the CB_1_R antagonist could promote fatty acid oxidation and energy uncoupling in adipose tissues (1). As well, adipocyte-specific deletion of the *CB1* gene was demonstrated to induce a lean phenotype in mice by promoting a thermogenic program in adipose tissue (37). In addition, in consistent with previous report in the diet-induced obese pre-diabetic mice (30), LH-21 also showed an overall metabolic improvement in glucose metabolism relevant parameters in the KKAy mice including ameliorated glucose intolerance and hyperinsulinemia, increased insulin sensitivity index, and a tendency to decrease fasting glucose; however, this may be realized through both the CB_1_R- and GPR55-mediated actions, as LH-21 was recently proved to be able to improve islet β-cell function and viability through directly activation of islet GPR55 (36).

Adipocyte CB_1_R plays crucial roles in controlling adipocyte physiology and in regulating systemic energy metabolism (37). The volume of adipose tissues enlarged aggressively under condition of morbid obesity and its function also will be dysregulated. Studies on animals and humans had revealed close connection between obesity and a state of low-grade, chronic inflammation characterized by increased circulating levels of pro-inflammatory molecules, including cytokines, adipokines, and chemokines (14, 18, 38, 39). The persistent low-grade activation of chronic inflammatory response in adipose tissue plays critical roles in the development of obesity-related hypertension and insulin resistance (16, 38). Correspondingly, in current study, the increased low-grade inflammation in inner of aortae (elevated pro-inflammatory cytokines TNFα, MCP-1, and ICAM-1) underpinned at least some of the pathological basis of the hypertension of the KKAy mice. Elevated accumulation of lipocalin-2 in intima layer of the aortic wall may contribute greatly in this process. Meanwhile, augmented exposure of the blood vessels to circulating inflammatory cytokines (TNFα, IL-6, and CXCL1) that secreted by deregulated adipose tissue also induces the endothelial dysfunction and oxidative stress of the aortic wall, and will further aggravate the development of systemic hypertension. Therefore, an efficient therapeutic strategy to treat metabolic disorders associated with obesity will contribute to ameliorate the dysregulated production of oxidative stress, inflammatory cytokines, and adipokines in adipose tissue. Herein, 3-week LH-21 treatment significantly decreased blood pressure in KKAy mice. From the view of possible pleiotropic effects of LH-21 on adipose tissue function, three interesting findings may contribute to its antihypertension effect: (1) reduced adipose tissue mass and improved energy metabolism, (2) a suppressive effect on gene expression and secretion of inflammatory cytokines, and (3) an ameliorating effect on adipokines production and secretion, particularly on lipocalin-2.

As one of the largest endocrine organs in the body, adipose tissue produces and secretes numerous adipokines, which play critical roles in obesity-related metabolic disorders (16, 40, 41). Lipocalin-2 is an inflammatory marker closely related to obesity, insulin resistance, and obesity-related hypertension (17, 19, 21). Adipose tissue is presumed to be the major source that contributed to the elevated circulating level of Lipocalin-2 in these pathological states (42). Serum and WAT concentration of lipocalin-2 had been found increased markedly in dietary and genetically obese animals with hypertension (19), whereas the obese mice deficient of lipocalin-2 exhibited significantly lower blood pressure (22). Meanwhile, the association between single-nucleotide polymorphisms in the gene encoding lipocalin-2 in humans also revealed a causal relationship between lipocalin-2 and development of hypertension. Moreover, the accumulation of lipocalin-2 protein in aortae of dietary obese mice with hypertension is also proved to be augmented significantly (22). Lipocalin-2 causes vascular inflammation, endothelial dysfunction, and finally hypertension by promoting oxidative stress and inflammatory reaction (22). In the KKAy mice, we also revealed elevated circulating lipocalin-2, and enhanced accumulation of lipocalin-2 in the aortae. However, the mRNA expression of lipocalin2 in the aortae is too low to be detected, this to some extent further indicated that lipocalin2 was not originated from the aortae, but from the adipose tissue. More importantly, gene and protein expression of lipocalin-2 in the WAT were found upregulated strikingly. This explained why the marker of inflammation in the aortae and blood pressure is increased in KKAy mice. LH-21 treatment effectively reversed the deleterious effect of lipocalin-2 by downregulating the expression and secretion of lipocalin-2 from adipose tissue, and thus lowered the blood pressure. However, the underlying modulatory mechanism to lipocalin-2 by LH-21 in adipose tissue still needs further detailed study. To clarify whether the effect was secondary to the amelioration of dysregulated metabolism in adipose tissue or it was a direct regulation.

Taken together, our current study demonstrated that the peripheral CB_1_R antagonist LH-21 is effective in ameliorating obesity-accompanied high blood pressure in the KKAy mice model of obesity and diabetes, and concomitantly improves systemic glucose handling and dyslipidemia. The underlying mechanism is closely in association with the amelioration on augmented levels and expression of pro-inflammatory cytokines and adipokines in serum and adipose tissues (Figure S1 in Supplementary Material). Particularly, alleviated circulating lipocalin-2 and its accumulation in aortae, and then vascular derangement and arterial hypertension was thus reversed. However, whether these benefits are being mediated exclusively by targeting the CB_1_R needs further demonstration, as LH-21 was demonstrated to be able to activate another GPCR, the GPR55 receptor, in modulating islet hormone secretion and reverting obesity-induced anxiety (30, 36).

## Ethics Statement

All animal handling and experiments were performed strictly in accordance with the recommendations of the Guide for the Care and Use of Laboratory Animals of the National Institutes of Health. The experimental protocol was approved by the Animal Experimental Ethics Committee of the FuDan University.

## Author Contributions

Conceived and designed the experiments: ZD, HG and XS. Performed the experiments: ZD, HW, YC and JW. Analyzed the data: ZD, HW, YC and YD. Wrote the paper: ZD and XS. Revised the paper: HG and XS.

## Conflict of Interest Statement

The authors declare that the research was conducted in the absence of any commercial or financial relationships that could be construed as a potential conflict of interest.

## Supplementary Material

Figure S1 Mechanistic scheme for the regulation of LH-21 on antihypertension in KKAy mice. BBB, blood–brain barrier. Red arrows indicate results from current study, and blue arrows indicate conclusions from literature.
