# Supplementary figures and images for: LH-21, A Peripheral Cannabinoid Receptor 1 Antagonist, Exerts Favorable Metabolic Modulation Including Antihypertensive Effect in KKAy Mice by Regulating Inflammatory Cytokines and Adipokines on Adipose Tissue
Source: Front Endocrinol (Lausanne). 2018 Apr 20;9:167. doi: 10.3389/fendo.2018.00167 (PMC5920035; doi:10.3389/fendo.2018.00167)

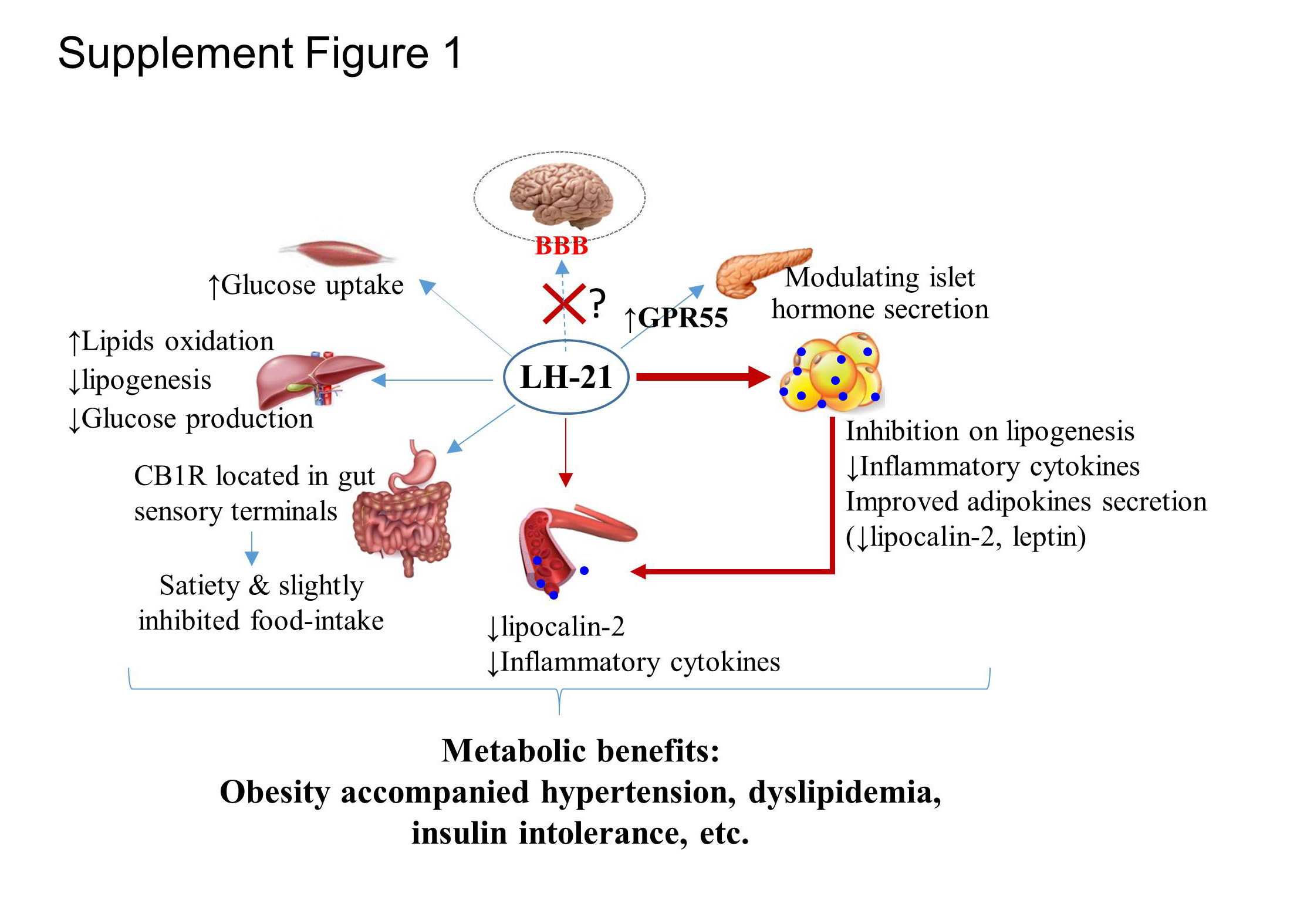

Supplement: Figure S1 — Mechanistic scheme for the regulation of LH-21 on antihypertension in KKAy mice. BBB, blood–brain barrier. Red arrows indicate results from current study, and blue arrows indicate conclusions from literature. [file image_1.JPEG]
