# Supplementary material for: LH-21, A Peripheral Cannabinoid Receptor 1 Antagonist, Exerts Favorable Metabolic Modulation Including Antihypertensive Effect in KKAy Mice by Regulating Inflammatory Cytokines and Adipokines on Adipose Tissue
Source: Front Endocrinol (Lausanne). 2018 Apr 20;9:167. doi: 10.3389/fendo.2018.00167 (PMC5920035; doi:10.3389/fendo.2018.00167)
Supplement: Supplementary file 2 [file table_1.DOC]

**Supplementary table 1. Primer sequences used for qPCR**

| ***Gene*** | **Forward primer** | **Reverse primer** |
| --- | --- | --- |
| *TNFa* | TGGTGCCTGGTCTGATGATG | GTGGTAACCGCTCAGGTGTTG |
| *MCP-1* | GCCTGCTGTTCACAGTTGC | GGTGATCCTCTTGTAGCTCTCC |
| *CXCL1* | TACCCAACCTTGGCTAGACG | GTCCGAGGAGAGAGCTTGC |
| *IL-6* | TGCTCTTCTGTATCGCCCAGT | GCCGTGTTAAGGAATCTGCTG |
| *PAI-1* | CAGTGTGGTGCACGTCTCCAAT | TGAACCAAAGTTGACCACCAG |
| *ICAM-1* | GTGATGCTCAGGTATCCATCCA | CACAGTTCTCAAAGCACAGCG |
| *Selectin* | ATGCCTCGCGCTTTCTCTC | CACAGTTCTCAAAGCACAGCG |
| *Leptin* | GAACCATGAAGCCAACGACT | GCGAAGTTCACAGTGGTTCC |
| *adiponectin* | TGTTGGAATGACAGGAGCTGA | CACACTGAACGCTGAGCGATAC |
| *Lipocalin-2* | GGTTTGGTGGCAGGCTATTA | CAGAGTGGCTTTCCCCATAA |
| *gp91phox* | TTTGTCAAGTGCCCCAAGGT | GGCATCTTGGAACTCCTGCT |
| *P22phox* | GGAGCGATGTGGACAGAAGT | GGCTGCCAGCAGATAGATCA |
| *P47phox* | ACATCACAGGCCCCATCATCCT | ACCCACCTCGCTTTGTCTTC |
| *β-actin* | TAAAGACCTCTATGCCAACAC | CACGATGGAGGGGCCGGACTC |
